# The transcriptome of peripheral blood mononuclear cells in patients with clinical subtypes of late age-related macular degeneration

**DOI:** 10.1186/s12979-019-0160-0

**Published:** 2019-08-15

**Authors:** Yousif Subhi, Marie Krogh Nielsen, Christopher Rue Molbech, Charlotte Liisborg, Helle Bach Søndergaard, Finn Sellebjerg, Torben Lykke Sørensen

**Affiliations:** 1 Clinical Eye Research Division, Department of Ophthalmology, Zealand University Hospital, Vestermarksvej 23, DK-4000 Roskilde, Denmark; 2 Faculty of Health and Medical Sciences, University of Copenhagen, Copenhagen, Denmark; 3 Danish Multiple Sclerosis Center, Copenhagen University Hospital Rigshospitalet, Copenhagen, Denmark

**Keywords:** Age-related macular degeneration, Choroidal neovascularization, Geographic atrophy, Polypoidal choroidal vasculopathy, Subretinal fibrosis, Peripheral blood mononuclear cells, Transcriptome

## Abstract

**Background:**

Peripheral blood mononuclear cells (PBMCs) are implicated in the pathogenesis of age-related macular degeneration (AMD). We here mapped the global gene transcriptome of PBMCs from patients with different clinical subtypes of late AMD.

**Results:**

We sampled fresh venous blood from patients with geographic atrophy (GA) secondary to AMD without choroidal neovascularizations (*n* = 19), patients with neovascular AMD without GA (*n* = 38), patients with polypoidal choroidal vasculopathy (PCV) (*n* = 19), and aged control individuals with healthy retinae (*n* = 20). We isolated PBMCs, extracted RNA, and used microarray to investigate gene expression. Volcano plots identified statistically significant differentially expressed genes (*P* < 0.05) at a high magnitude (≥30% higher/lower) for GA (62 genes), neovascular AMD (41 genes), and PCV (41 genes). These clinical subtypes differed substantially across gene expression and the following pathways identified in enrichment analyses. In a subgroup analysis, we investigated presence vs. absence of subretinal fibrosis and found 826 differentially expressed genes (≥30% higher/lower, *P* < 0.05) with relation to mRNA splicing, endothelial migration, and interleukin-1 signaling.

**Conclusions:**

We here map the global gene transcriptome of PBMCs related to clinical subtypes of late AMD and find evidence of subtype-specific immunological involvement. Our findings provide a transcriptomic insight into the systemic immunity associated with AMD.

**Electronic supplementary material:**

The online version of this article (10.1186/s12979-019-0160-0) contains supplementary material, which is available to authorized users.

## Background

Age-related macular degeneration (AMD) is the most frequent cause of visual impairment in the developed countries and the demographic developments towards an aging population are expected to continually and significantly increase the disease burden in the years to come [1–3]. Early stages of the disease are characterized by drusen formation. Drusen are deposits of extracellular material and lipoproteins under the retinal pigment epithelium (RPE) that to a certain extent are considered a normal aging phenomenon, and they are typically asymptomatic [4, 5]. While this stage characterizes the majority of individuals with AMD, an important group of patients experience progression of disease to the late form of AMD [4].

Late AMD manifests with different clinical features. In geographic atrophy (GA), fundus examination reveals the demarcated areas of atrophy of the neuroretina and the RPE, which gradually progress over time. This subtype of late AMD, GA without any choroidal neovascularizations (CNV), accounts for ~ 40% of all late AMD cases in Caucasian populations [3, 4, 6]. Currently, no treatment exists [4]. This is in contrast to neovascular AMD, where CNVs development is mediated by vascular endothelial growth factor (VEGF) expression, which is treatable through regular intravitreal injections with anti-VEGF antibodies [4].

When diagnosing neovascular AMD, retinal angiography is the gold standard and polypoidal choroidal vasculopathy (PCV) is a frequent differential diagnosis [7–9]. PCV is present in ~ 9% of White patients and ~ 50% of Asian patients suspected of neovascular AMD who undergo retinal angiography for diagnosis [7]. Interestingly, PCV is not strongly associated to drusen as otherwise observed in eyes with neovascular AMD [7], and studies increasingly deal with the question of whether PCV is just another clinical phenotype of neovascular AMD or a distinct clinical entity [10].

Intravitreal injection treatment with anti-VEGF has dramatically improved the prognosis of neovascular AMD [11, 12]. However, when left untreated, on average 1 line (defined as five letters constituting a line on the Early Treatment of Diabetic Retinopathy Study visual acuity chart) is lost at 3 months, 3 lines are lost at 12 months, and 4 lines are lost at 24 months; mainly due to progression of the neovascular lesion and through development of fibrotic scars [13]. In the era of modern anti-VEGF therapy, subretinal fibrosis is considered a harbinger of poor visual outcomes [14]. It may be present at time of diagnosis or develop gradually despite anti-VEGF treatment [14]. There is currently no treatment for subretinal fibrosis.

The exact pathogenesis of late AMD remains to be mapped, but consensus so far is that late AMD is a complex interplay of susceptibility through genetic background, chronic progressive degeneration of the macula, immunosenescence, and dysfunction of the immune system [4, 5, 15–19]. Emerging evidence suggest correlations between specific pathological mechanisms and different clinical phenotypes of late AMD [20–30]. Involvement of systemic immune cells is demonstrated in an increasing number of observational studies in human patients with clinical subtypes of late AMD [20–31]. In this study, we investigated the global gene transcriptome of peripheral blood mononuclear cells (PBMC) across different clinical subtypes of late AMD and compared to those in aged control individuals with healthy retinae. We also compared individuals with any subretinal fibrosis to those without subretinal fibrosis. This comparison provided important insight into a currently untreatable clinical subtype of neovascular AMD. Overall, we find that the PBMC in patients with late AMD differ from healthy controls, but that specific clinical subtypes associate with specific immunological changes.

## Methods

### Study design and ethics

This was a prospective clinic-based case-control study. We explained the nature of the study to potential participants prior to any participation and all participants gave oral and written informed consent. All aspects of this study follow the principles in the Declaration of Helsinki and ethical approval was obtained by the Regional Committee of Ethics in Research of the Region of Zealand (SJ-379).

### Study participants and eligibility

Patients were recruited from our outpatient retinal clinic at Department of Ophthalmology, Zealand University Hospital, Roskilde, Denmark. We included three clinical subtypes of late AMD and one aged control group with healthy retina in both eyes. Participants with late AMD had: 1) GA secondary to AMD in one or both eyes with no signs of CNV, 2) neovascular AMD in one or both eyes with no signs of GA, 3) PCV in one or both eyes with no GA. Among patients with neovascular AMD, we included equally cases of patients who had no subretinal fibrosis in both eyes and patients who had subretinal fibrosis in one or both eyes. We did not include patients with recent onset of CNV because we previously found indication of possible acute immune activity in relation to recent onset of CNV [32]. In addition, we wanted to evaluate whether a patient would develop subretinal fibrosis, which would be difficult to evaluate in a treatment-naïve eye. Elderly healthy control individuals were recruited among biologically unrelated healthy relatives to the patients. This was an intentional strategy applied to better match the control group on environmental exposures.

We employed two different strategies in participant selection to avoid influence of non-AMD related immunology. Participants were only included if they did not have any active cancer, autoimmune disease, or any active immune, infectious or inflammatory diseases, or were in chemotherapy or immunomodulating therapy for any reason. We sampled blood from potential participants in lithium-heparin coated tubes for routine plasma C-reactive protein measurement and did not include any participant with plasma C-reactive protein > 15 mg/L, which is a sign of ongoing acute immune response [33].

To evaluate number of participants needed, we looked at the literature of the transcriptome of the PBMC. Grunin et al. were able to successfully evaluate the transcriptome of blood monocytes based on a sample of 14 patients with neovascular AMD and 15 healthy controls [34]. Thus, we aimed for 96 participants in total (analyses are made in four batches with 24 samples each) with at least 15 individuals in each group (patients with GA with no CNV; patients with neovascular AMD with no GA and no subretinal fibrosis; patients with neovascular AMD with no GA but with subretinal fibrosis; patients with PCV; healthy controls).

### Retinal diagnosis and clinical data

Participants were examined using slit-lamp bio-microscopy and fundus examination, scanning laser imaging, spectral-domain optical coherence tomography (OCT), fundus autofluorescence, and retinal angiography (fluorescein and indocyanine green angiography (ICGA)) where CNV was suspected. We used the following definitions for participant grouping:

- Healthy controls: participants with healthy retinae defined as less than 10 small drusen (diameter < 63 μm); no signs of choroidal abnormalities, atrophy, CNV, or pigment abnormalities; and no signs of other retinal diseases.
- Patients with GA: participants who had GA secondary to AMD in one or both eyes without any signs of CNV. This was defined as a presence of drusen maculopathy with one or more well-defined atrophic area(s) with decreased retinal pigment seen on OCT corresponding to hypofluorescence on fundus autofluorescence.
- Patients with neovascular AMD: participants who had fibrovascular detachment of the retinal pigment epithelium and choroidal neovascular membranes with subretinal or sub-RPE hemorrhages or fibrosis, and no signs of GA. Presence of subretinal fibrosis was evaluated on OCT and used for subgrouping into those with either no subretinal fibrosis in both eyes or those with any subretinal fibrosis in one or both eyes.
- Patients with PCV: participants who had one or more polyps in early-phase ICGA with a hypofluorescent halo and with/without branching vascular networks, and without any GA. Other PCV stigmata used to support the diagnosis of PCV were orange-red focal subretinal polyp-like structures, pulsation of polyps on ICGA video, and a protrusion from the choroid elevating RPE from the Bruch’s membrane observed on OCT.

All participants were interviewed to obtain data on lifestyle and medical history. Medical data were crosschecked with the electronic patient record. Smoking habits were categorized in current smokers, previous smokers (smoked > 100 cigarettes during lifetime and ceased smoking > 12 months), or never smokers. Alcohol use was reported in units/week (1 unit = 12 mL ethanol), which is a measure widely used in Denmark by layman. Physical activity was assessed using a single question on regular activity validated in previous studies for patients with AMD in Denmark [33, 35]. Height and weight were measured to calculate body mass index (BMI).

### Tissue sampling and preparation

Venous blood (10 mL) was sampled from antecubital veins in ethylenediaminetetraacetic acid (EDTA) coated tubes. The EDTA stabilized blood was prepared within 4 h. In a 15 mL centrifuge tube, we added 5 mL LymphoPrep™ (STEMCELL Technologies Inc., Vancouver, British Columbia, Canada) and then 10 mL blood carefully on top of the LymphoPrep layer. Lymphoprep™ is a density gradient medium that allows isolation of PBMC through centrifugation. We centrifuged the tube for 30 min at 4 °C at 1000 *g* with slow acceleration and deceleration. This process allowed following separation (in order from top to bottom): plasma, PBMCs, LymphoPrep, granulocytes, and erythrocytes. We carefully transferred the PBMC layer into a 2 mL tube, centrifuged (5 min at 4 °C at 1000 *g*), removed the supernatant, added 1.5 mL 4 °C sterile phosphate-buffered saline, centrifuged (5 min at 4 °C at 1000 *g*), removed the supernatant, and snap-froze the remaining pellet in liquid nitrogen where it was stored until ribonucleic acid (RNA) extraction and analysis. Transportation to RNA extraction was made using dry ice.

### Microarray analysis, bioinformatics, and statistical analysis

RNA was extracted from PBMC using NuGEN Ovation® Pico WTA System V2 kit (NuGEN Technologies Inc., Redwood City, CA, USA) according to the manufacturer’s recommendations. The RNA was analyzed for gene expression with the Human Gene 2.0 ST array (Affymetrix, Santa Clara, CA, USA), which contains 1–2 probes/exon and ~ 26 probes/transcript in total containing 11,086 long intergenic non-coding RNA transcripts and a RefSeq gene count of 24,838. The RNA was labelled using the NuGen Ovation Kit (NuGEN Technologies, San Carlos, California, USA). The arrays were washed and strained with phycoerytrin-conjugated streptavidin using the Affymetrix Fluidics Station® 450 and the arrays were scanned in the Affymetrix GeneArray® 3000 scanner to generate fluorescent images, as described in the Affymetrix GeneChip® protocol. Cell intensity files (CEL files) were generated in the Affymetrix GeneChip® Commond Console® Software.

Participant characteristics were summarized using descriptive statistics and compared using parametric statistics or non-parametric statistics according to distribution characteristics of the variables. Categorical variables were summarized using numbers and percentages and compared using χ^2^-test or Fisher’s Exact test when categories were small. Statistics were made using SPSS 23 (IBM, Armonk, New York, USA). A *p*-value less than 0.05 was considered statistically significant. Microarray data are modelled using the Robust Multichip Average approach followed by mean one-step probe set summarization giving each gene a single expression value, all done using the software package Partek Gemonics Suite 6 (Partek, St. Louis, Missouri, USA). Gene annotation list is available as Additional file 1. Comparisons between groups were made using an analysis of covariance (ANCOVA) including run date, age, and sex as covariates and with clinical disease subtype versus healthy control individuals as contrast. For subgroup analyses comparing patients with neovascular AMD with or without subretinal fibrosis, we performed new ANCOVA analysis including run date, age, and sex as covariates and presence of subretinal fibrosis vs. absence of subretinal fibrosis as contrast. Differentially expressed genes were defined as those expressed either ≥30% or ≤ − 30% in a disease group compared to the healthy control individuals and where statistically significant. Volcano plots were used to illustrate differences between the groups. Heatmaps and hierarchical clustering were made using Partek Gemonics Suite 6. Differentially expressed genes were summarized in tables and analyzed using the Enrichr database (http://amp.pharm.mssm.edu/Enrichr/) to predict functional pathways and biological functions of the differentially expressed genes [36, 37].

## Results

We included a total of 96 participants: 19 patients with GA with no CNV, 38 patients with neovascular AMD with no GA, 19 patients with PCV, and 20 healthy controls. Participant characteristics are summarized in Table 1. We obtained gene expression values from a total of 29,410 genes

**Table 1:** Participant characteristics

	Patients with GA (*n* = 19)	Patients with nAMD (*n* = 38)	Patients with PCV (*n* = 19)	Healthy controls (*n* = 20)	*P*-value
Age, years, mean (SD)	80.4 (8.3)	78.3 (7.8)	71.9 (7.8)	71.7 (8.9)	0.001
Females, n (%)	8 (42)	17 (45)	13 (68)	14 (70)	0.11
Smoking status, n (%)					0.50
Active	5 (26)	10 (26)	7 (37)	3 (15)	
Ex-smoker	10 (53)	7 (18)	3 (16)	8 (40)	
Never smoker	4 (21)	21 (56)	9 (47)	9 (45)	
Alcohol consumption, units, median (IQR)	3 (2 to 10)	7 (1 to 10)	4 (1 to 13)	5 (1 to 8)	0.96
Body mass index, mean (SD)	26.5 (6.7)	25.3 (4.4)	25.2 (3.1)	24.7 (3.1)	0.68
Physically active, n (%)	11 (58)	21 (55)	10 (53)	12 (60)	0.97

*Abbreviations*: *GA* Geographic atrophy, *IQR* Interquartile range, *nAMD* Neovascular age-related macular degeneration, *PCV* Polypoidal choroidal vasculopathy, *SD* Standard deviation

### Differentially expressed genes in PBMCs across clinical subtypes of late AMD

**Fig. 1:**
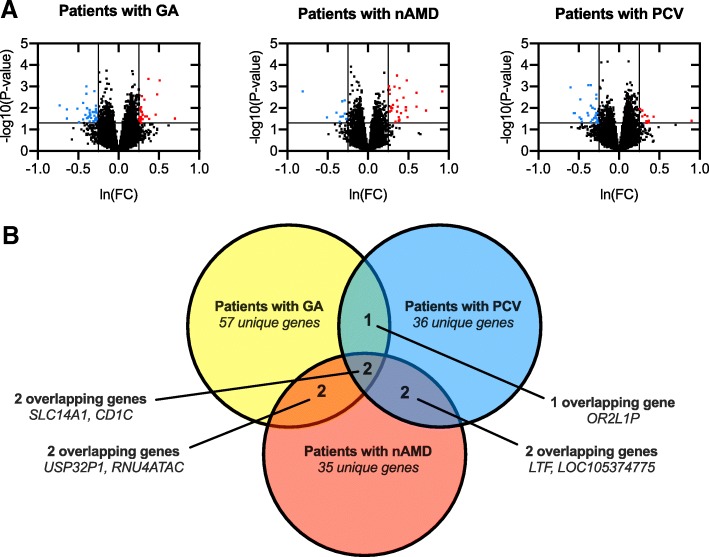
Volcano plots of all quantified genes in the transcriptome analysis of peripheral blood mononuclear cells (PBMCs) and Venn diagram to illustrate similarities and differences. **a** Volcano plots the PBMC transcriptome for each of the different clinical subtype of late AMD when compared to the healthy controls. Significantly differentially expressed genes are defined as those with at least ±30% change (level of magnitude, vertical lines) and *P* < 0.05 (level of statistical significance, horizontal line) and illustrated in red (increased expression) or blue (decreased expression). *P*-values are obtained using analysis of covariance including run date, age, and sex as co-variates and with patients versus healthy controls as contrast. *P*-values are logarithmic transformed for plot construction. **b** Venn diagram illustrates the small overlap of differentially expressed genes across the different clinical subtypes

The total transcriptome across different clinical subtypes and heatmaps are available as Additional files 2 and 3. Volcano-plots illustrate differentially expressed genes across different clinical subtypes of late AMD (Fig. 1). In patients with GA, 817 (3%) genes were differentially expressed (*P* < 0.05); of these, 27 genes had ≥30% higher and 35 genes had ≥30% lower expression (Table 2). In patients with neovascular AMD, 644 (2%) genes were differentially expressed (*P* < 0.05); of these, 31 genes had ≥30% higher and 10 had ≥30% lower expression (Table 2). In patients with PCV, 806 (3%) genes were differentially expressed (*P* < 0.05); of these, 11 genes had ≥30% higher and 30 genes had ≥30% lower expression (Table 2). These significantly (both in terms of statistics and magnitude) differentially expressed genes overlapped to a minor degree and at a higher degree between GA and nAMD and between nAMD and PCV than between GA and PCV (Fig. 1).

**Table 2:** Significantly differentially expressed genes in peripheral blood mononuclear cells of patients with different clinical subtypes of late AMD

Gene name	Description	FC %	*P*-value
Patients with GA			
SNORA14A	small nucleolar RNA, H/ACA box 14A	101	0.031
USP32P1	ubiquitin specific peptidase 32 pseudogene 1	67	< 0.001
SLC14A1	solute carrier family 14 (urea transporter), member 1 (Kidd blood group)	62	0.002
MIR4516	microRNA 4516	59	0.020
DDA1	DET1 and DDB1 associated 1	46	0.033
MIR3665	microRNA 3665	44	< 0.001
SNORA49	small nucleolar RNA, H/ACA box 49	43	0.025
MIR4509–1	microRNA 4509–1	39	0.024
TMEM120A	transmembrane protein 120A	38	0.004
LY6E	lymphocyte antigen 6 complex, locus E	35	0.021
IFITM1	interferon induced transmembrane protein 1	35	0.033
CSNK1G2	casein kinase 1, gamma 2	34	0.015
NINJ1	ninjurin 1	33	0.003
HSH2D	hematopoietic SH2 domain containing	33	0.009
GIMAP1-GIMAP5	GIMAP1-GIMAP5 readthrough	32	0.030
LINC01000	long intergenic non-protein coding RNA 1000	32	0.026
SNORD88A	small nucleolar RNA, C/D box 88A	32	0.014
CAMKK2	calcium/calmodulin-dependent protein kinase kinase 2, beta	32	0.049
KRT73	keratin 73, type II	32	0.034
SDF4	stromal cell derived factor 4	31	0.012
CD247	CD247 molecule	31	0.042
TMSB4XP4	thymosin beta 4, X-linked pseudogene 4	31	0.026
CD200R1	CD200 receptor 1	31	0.032
CISH	cytokine inducible SH2-containing protein	31	0.011
MX2	MX dynamin-like GTPase 2	30	0.035
MICALCL	MICAL C-terminal like	30	0.026
CD5	CD5 molecule	30	0.037
C15orf54	chromosome 15 open reading frame 54	− 108	0.008
RNU4ATAC	RNA, U4atac small nuclear (U12-dependent splicing)	− 91	0.031
LOC105370195	uncharacterized LOC105370195	− 89	0.012
MYCT1	myc target 1	−67	0.011
MS4A1	membrane-spanning 4-domains, subfamily A, member 1	−64	0.045
LOC105377384	uncharacterized LOC105377384	−56	0.034
OR2L1P	olfactory receptor, family 2, subfamily L, member 1 pseudogene	−55	0.006
LOC105379818	uncharacterized LOC105379818	−51	0.021
CD1C	CD1c molecule	−49	0.001
LOC105369884	uncharacterized LOC105369884	−49	0.002
LOC102724714	uncharacterized LOC102724714	−47	0.030
RSU1	Ras suppressor protein 1	−47	0.010
TNFSF4	tumor necrosis factor (ligand) superfamily, member 4	−46	0.028
SNORD42A	small nucleolar RNA, C/D box 42A	−45	0.026
WDR11-AS1	WDR11 antisense RNA 1	− 43	0.025
LINC00989	long intergenic non-protein coding RNA 989	−43	0.043
CRYZ	crystallin zeta	−43	0.016
DNM3	dynamin 3	−43	0.012
LINC00266–1	long intergenic non-protein coding RNA 266–1	−42	0.021
SLFN14	schlafen family member 14	−40	0.029
HIST1H2BC	histone cluster 1, H2bc	−38	0.016
GFI1B	growth factor independent 1B transcription repressor	−37	0.016
PF4	platelet factor 4	−37	0.015
PPIAL4A	peptidylprolyl isomerase A (cyclophilin A)-like 4A	−36	0.041
SENCR	smooth muscle and endothelial cell enriched migration/differentiation-associated lncRNA	−36	0.002
LOC101928979	uncharacterized LOC101928979	−35	0.023
MIR1321	microRNA 1321	−34	0.016
CTTN	cortactin	−33	0.033
GRB14	growth factor receptor bound protein 14	−32	0.008
PCYT1B	phosphate cytidylyltransferase 1, choline, beta	−32	0.028
P2RY1	purinergic receptor P2Y, G-protein coupled, 1	−32	0.011
KCNQ5-IT1	KCNQ5 intronic transcript 1	−32	0.037
FAM111B	family with sequence similarity 111, member B	−32	0.014
GUSBP1	glucuronidase, beta pseudogene 1	−31	0.033
LIMS1	LIM and senescent cell antigen-like domains 1	−31	0.016
Patients with nAMD			
OSBP2	oxysterol binding protein 2	151	0.002
IGHV1OR21–1	immunoglobulin heavy variable 1/OR21–1 (non-functional)	106	0.013
ANK1	ankyrin 1, erythrocytic	83	0.002
PITHD1	PITH (C-Terminal Proteasome-Interacting Domain Of Thioredoxin-Like) Domain Containing 1	82	0.009
MIR4644	microRNA 4644	64	0.027
MARCH8	membrane associated ring finger 8	61	0.001
PAGE2B	P antigen family, member 2B	60	0.009
TMOD1	tropomodulin 1	60	0.013
HBD	hemoglobin, delta	56	0.003
LOC401127	WD repeat domain 5 pseudogene	51	0.018
RHD	Rh blood group, D antigen	48	0.036
TRBV7–4	T cell receptor beta variable 7–4 (gene/pseudogene)	48	0.001
USP32P1	ubiquitin specific peptidase 32 pseudogene 1	47	0.038
FAM210B	family with sequence similarity 210, member B	47	0.027
BPGM	2,3-bisphosphoglycerate mutase	47	0.040
FECH	ferrochelatase	45	0.009
LINC01291	long intergenic non-protein coding RNA 1291	44	< 0.001
HBM	hemoglobin, mu	43	0.015
MMP8	matrix metallopeptidase 8	43	0.004
DCAF12	DDB1 and CUL4 associated factor 12	41	0.044
FAM46C	family with sequence similarity 46, member C	40	0.043
LTF	lactotransferrin	39	0.001
PGLYRP1	peptidoglycan recognition protein 1	37	0.007
CAMP	cathelicidin antimicrobial peptide	35	0.010
SLC25A39	solute carrier family 25, member 39	34	0.010
SLC14A1	solute carrier family 14 (urea transporter), member 1 (Kidd blood group)	34	0.013
SLC25A37	solute carrier family 25 (mitochondrial iron transporter), member 37	32	0.009
SLC4A1	solute carrier family 4 (anion exchanger), member 1 (Diego blood group)	31	0.001
IFIT1B	interferon-induced protein with tetratricopeptide repeats 1B	31	0.002
AHSP	alpha hemoglobin stabilizing protein	31	0.015
ALAS2	5-aminolevulinate synthase 2	30	0.001
RNU4ATAC	RNA, U4atac small nuclear (U12-dependent splicing)	− 125	0.002
SNORD3C	small nucleolar RNA, C/D box 3C	−66	0.028
ADAM28	ADAM metallopeptidase domain 28	− 48	0.017
RNU5E-1	RNA, U5E small nuclear 1	−42	0.044
SNORD12C	small nucleolar RNA, C/D box 12C	−41	0.026
FRG1JP	FSHD region gene 1 family member J, pseudogene	−37	0.005
MIR1537	microRNA 1537	−36	0.025
CD1C	CD1c molecule	−34	0.004
SNAR-G2	small ILF3/NF90-associated RNA G2	− 34	0.018
LOC105374775	uncharacterized LOC105374775	−31	0.033
Patients with PCV			
TRIM48	tripartite motif containing 48	146	0.040
LOC105373103	uncharacterized LOC105373103	54	0.026
TRAJ13	T cell receptor alpha joining 13	45	0.046
SLC14A1	solute carrier family 14 (urea transporter), member 1 (Kidd blood group)	44	0.040
IGKV1–12	immunoglobulin kappa variable 1–12	42	0.024
LTF	lactotransferrin	41	0.050
TRAV8–4	T cell receptor alpha variable 8–4	40	0.041
IGHG4	immunoglobulin heavy constant gamma 4 (G4 m marker)	37	0.021
IGKV2D-24	immunoglobulin kappa variable 2D-24 (non-functional)	34	0.024
SNORA71D	small nucleolar RNA, H/ACA box 71D	34	0.013
FAR2P3	fatty acyl-CoA reductase 2 pseudogene 3	30	0.011
LOC101928215	uncharacterized LOC101928215	−81	0.001
SNORD117	small nucleolar RNA, C/D box 117	− 76	0.017
SNORD63	small nucleolar RNA, C/D box 63	−73	0.017
SCARNA9L	small Cajal body-specific RNA 9-like	−63	0.028
SNORD14E	small nucleolar RNA, C/D box 14E	−62	0.004
SNORD59B	small nucleolar RNA, C/D box 59B	−61	0.032
ZEB2	zinc finger E-box binding homeobox 2	−58	0.044
LOC105370515	uncharacterized LOC105370515	−48	0.017
H1F0	H1 histone family, member 0	−47	0.017
USP12-AS1	USP12 antisense RNA 1	−47	0.023
TRAJ56	T cell receptor alpha joining 56	−46	0.001
CD1C	CD1c molecule	−42	0.001
RNVU1–15	RNA, variant U1 small nuclear 15	−42	0.008
LOC728093	putative POM121-like protein 1-like	−41	0.045
LOC105374775	uncharacterized LOC105374775	−40	0.010
LOC105375566	uncharacterized LOC105375566	−39	0.035
LOC101927770	uncharacterized LOC101927770	−36	0.030
FLT3	fms-related tyrosine kinase 3	−35	0.011
OR2L1P	olfactory receptor, family 2, subfamily L, member 1 pseudogene	−34	0.030
LOC100272216	uncharacterized LOC100272216	−34	0.028
MERTK	MER proto-oncogene, tyrosine kinase	−34	0.023
MIR486–2	microRNA 486–2	−33	0.029
NFKBIZ	nuclear factor of kappa light polypeptide gene enhancer in B-cells inhibitor, zeta	−33	0.046
LOC105378488	uncharacterized LOC105378488	−33	0.017
MIR3160–1	microRNA 3160–1	−32	0.004
YPEL5	yippee like 5	−32	0.002
NLRP3	NLR family, pyrin domain containing 3	−31	0.035
SNORA7B	small nucleolar RNA, H/ACA box 7B	−31	0.045
HIST1H2BN	histone cluster 1, H2bn	−31	0.018
LOC101929516	uncharacterized LOC101929516	−30	0.049

*Abbreviations*: *FC* Fold change, *GA* Geographic atrophy, *nAMD* Neovascular age-related macular degeneration, *PCV* Polypoidal choroidal vasculopathy

### Pathways analysis across clinical subtypes of late AMD

Identifying a set of genes with higher or lower expression do provide important data into mechanisms of disease, but their collective pattern also provides important insight of a general trend. One popular and powerful method is to analyze expression patterns to prior knowledge gene-set libraries—the gene set enrichment analysis approach. Using the Gene Ontology (GO) resource, it is possible to predict these functional pathways in the three domains: biological process, molecular function, and cellular component. Results are presented in Table 3 for each clinical subtype of late AMD. Interestingly, although GO Biological Process revealed involvement of the immune system in all three clinical subtypes, different pathways were predicted across the different clinical subtypes: GA was associated with pathways in type I interferon signaling and memory T cell differentiation; neovascular AMD was associated with pathways in oxygen gas homeostasis and T cell activation; and PCV was associated with pathways in immune regulation and T-helper 2 cell related functions.

**Table 3:** Enrichr-based Gene Ontology (GO) enrichment analysis of genes significantly increased or decreased in expression in peripheral blood mononuclear cells of patients with different clinical subtypes of late AMD. Listed are the three strongest in each category (ranked using the Enrichr computed combined score (CS) which multiplies log *p*-value to z-score; only CS > 5 were considered to only consider strong signals) within the three GO-terms: Biological Process (BP), Molecular Function (MF), and Cellular Component (CC)

Increased			Decreased		
GO	Description	CS	GO	Description	CS
Patients with GA vs. Healthy controls					
BP	response to interferon-alpha	15.10	BP	positive regulation of memory T cell differentiation	14.05
BP	positive regulation of autophagy of mitochondrion	13.71	BP	regulation of memory T cell differentiation	13.18
BP	type I interferon signaling pathway	13.08	BP	positive regulation of T-helper 2 cell differentiation	13.16
MF	urea transmembrane transporter activity	15.87	MF	NADPH binding	12.91
MF	acetylcholine receptor regulator activity	15.48	MF	G-protein coupled nucleotide receptor activity	12.90
MF	amide transmembrane transporter activity	12.40	MF	G-protein coupled purinergic nucleotide receptor activity	12.70
CC	T cell receptor complex	10.27	CC	–	–
CC	Cul4-RING E3 ubiquitin ligase complex	6.62	CC	–	–
CC	–	–	CC	–	–
Patients with nAMD vs. Healthy controls					
BP	oxygen gas homeostasis	21.30	BP	lymphocyte activation involved in immune response	10.65
BP	gas homeostasis	18.33	BP	T cell activation involved in immune response	8.71
BP	regulation of cellular carbohydrate catabolic process	17.35	BP	–	–
MF	urea transmembrane transporter activity	15.36	MF	peptidase activity, acting on L-amino acid peptides	5.27
MF	iron ion transmembrane transporter activity	15.27	MF	–	–
MF	sodium:bicarbonate symporter activity	13.78	MF	–	–
CC	specific granule lumen	26.32	CC	–	–
CC	tertiary granule lumen	25.98	CC	–	–
CC	endocytic vesicle lumen	20.12	CC	–	–
Patients with PCV vs. Healthy controls					
BP	regulator of immune effector process	18.39	BP	negative regulation of acute inflammatory response	13.62
BP	positive regulation of toll-like receptor 4 signaling pathway	18.14	BP	positive regulation of T-helper 2 cell differentiation	13.62
BP	negative regulation by host of viral process	15.92	BP	regulation of T-helper 2 cell cytokine production	12.56
MF	urea transmembrane transporter activity	18.92	MF	vascular endothelial growth factor-activated receptor activity	13.97
MF	amide transmembrane transporter activity	14.94	MF	MAP kinase kinase kinase activity	11.30
MF	signal recognition particle binding	13.89	MF	transmembrane receptor protein tyrosine kinase activity	9.35
CC	endocytic vesicle lumen	11.94	CC	NLRP3 inflammasome complex	9.85
CC	specific granule lumen	6.87	CC	nuclear euchromatin	9.33
CC	tertiary granule lumen	6.77	CC	euchromatin	8.98

*Abbreviations*: *BP* Biological process, *CC* Cellular component, *CS* Combined score, *GA* Geographic atrophy, *GO* Gene ontology, *MF* Molecular function, *nAMD* Neovascular age-related macular degeneration, *PCV* Polypoidal choroidal vasculopathy

### Differences in the PBMC transcriptome among patients with neovascular AMD between those with and without subretinal fibrosis

Characteristics of patients with neovascular AMD with (*n* = 21) or without subretinal fibrosis (*n* = 17) are summarized in Table 4. We observed a trend towards higher age in those with subretinal fibrosis, which did not reach statistical significance (mean difference: 4.7 years; 95% confidence interval: 9.9 to − 0.4 years; *P* = 0.071). However, patients with subretinal fibrosis had been followed in the retinal clinic for a significantly longer time than those without subretinal fibrosis (mean difference: 18 months; 95% confidence interval: 2 to 35 months; *P* = 0.028). These circumstances follow the general clinical experience that 1) subretinal fibrosis occur if time to diagnosis occur with delay—a problem among the very old Danes who seem more settled with poor vision because of age and do not always react upon subtle symptoms—and 2) subretinal fibrosis can develop with time despite treatment.

**Table 4:** Characteristics of patients with neovascular AMD stratified according to whether subretinal fibrosis was absent or present

	Subretinal fibrosis absent (*n* = 17)	Subretinal fibrosis present (*n* = 21)	*P*-value
Age, years, mean (SD)	75.7 (8.6)	80.4 (6.5)	0.071
Females, n (%)	7 (41)	10 (48)	0.69
Smoking status, n (%)			0.23
Active	5 (29)	5 (24)	
Ex-smoker	7 (29)	14 (10)	
Never smoker	5 (41)	2 (67)	
Alcohol consumption, units, median (IQR)	7 (1 to 14)	7 (1 to 10)	0.28
Body mass index, mean (SD)	25.1 (3.4)	25.5 (5.1)	0.75
Physically active, n (%)	8 (47)	13 (62)	0.36
Time from diagnosis to sampling^a^, mean (SD)	19 (14)	38 (33)	0.028

*Abbreviations*: *IQR* Interquartile range, *SD* Standard deviation

^a^In cases with bilateral disease, time from diagnosis to sampling was defined as the time from diagnosis of the first eye

**Fig. 2:**
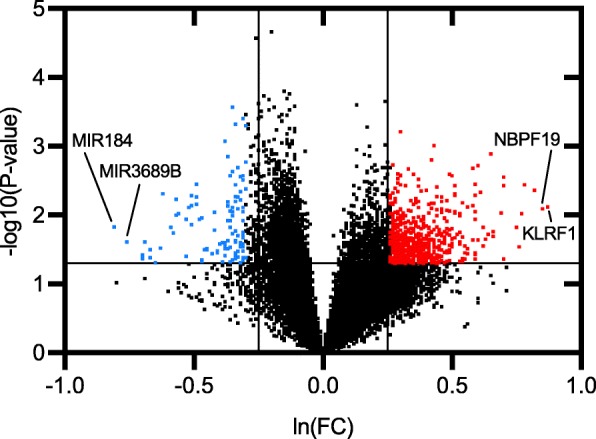
Volcano plots of all quantified genes in the transcriptome analysis of peripheral blood mononuclear cells of patients with neovascular AMD with subretinal fibrosis compared to those without. Significantly differentially expressed genes are defined as those with at least ±30% change (level of magnitude, vertical lines) and *P* < 0.05 (level of statistical significance, horizontal line) and illustrated in red (increased expression) or blue (decreased expression). The two genes with highest increase or decrease are stated with names. *P*-values are obtained using analysis of covariance including run date, age, and sex as co-variates and with patients with subretinal fibrosis versus patients without subretinal fibrosis as contrast. *P*-values are logarithmic transformed for plot construction

The gene list of differentially expressed genes between patients with neovascular AMD with or without subretinal fibrosis and heat-maps are available as Additional files 4 and 5. Volcano-plots illustrate these differentially expressed genes (Fig. 2). A total of 3344 (13%) genes were differentially expressed (*P* < 0.05); of these, 663 genes had ≥30% higher and 163 genes had ≥30% lower expression (see Additional file 6). Genes differentially expressed were included for enrichment analyses (Table 5). Enrichment analyses suggested the involvement of mRNA splicing mechanisms, regulation of interleukin-1 secretion, and endothelial cell migration.

**Table 5:** Enrichr-based Gene Ontology (GO) enrichment analysis of genes significantly increased or decreased in expression in peripheral blood mononuclear cells of patients with neovascular AMD with subretinal fibrosis compared to those without subretinal fibrosis. Listed are the three strongest in each category (ranked using the Enrichr computed combined score (CS) which multiplies log *p*-value to z-score; only CS > 5 were considered to only consider strong signals) within the three GO-terms: Biological Process (BP), Molecular Function (MF), and Cellular Component (CC)

Subretinal fibrosis present vs. Subretinal fibrosis absent					
Increased			Decreased		
GO	Description	CS	GO	Description	CS
BP	mRNA splicing, via spliceosome	12.17	BP	negative regulation of interleukin-1 beta secretion	6.71
BP	mRNA splice site selection	12.17	BP	endothelial cell migration	6.65
BP	regulation of mRNA splicing, via spliceosome	11.93	BP	negative regulation of interleukin-1 secretion	6.36
MF	ubiquitin-like protein ligase activity	29.03	MF	dipeptidyl-peptidase activity	7.76
MF	RNA binding	28.09	MF	caspase binding	7.07
MF	ubiquitin-specific protease binding	27.92	MF	–	–
CC	nuclear speck	21.62	CC	invadopodium	5.53
CC	nuclear body	21.52	CC	–	–
CC	ribonucleoprotein granule	14.72	CC	–	–

*Abbreviations*: *BP* Biological process, *CC* Cellular component, *CS* Combined score, *GO* Gene ontology, *MF* Molecular function

## Discussion

Defining clinical features of late AMD—choroidal neovascularization, geographically demarcated areas of atrophy, and choroidal polyps—illustrate the wide pathophysiological heterogeneity that is included in the term late AMD. Acknowledging this heterogeneity, studies are increasingly focusing on explaining disease mechanisms of these specific clinical features [20–31]. Peripheral immunological changes in human patients with AMD are gaining increasingly attention, and in this study, we shed important light into how different clinical features of late AMD correlate to the PBMC transcriptome. We find that only a small number of genes (< 0.1–0.2%) are differentially expressed at a high magnitude (≥ 30%) in patients with GA, neovascular AMD, and PCV, and that only a few genes overlap between these clinical subtypes. Our findings underscore the increasing notion that different mechanisms may be involved in these different clinical subtypes.

Pathophysiological mechanisms of GA are incompletely understood, but a range of findings in the immune system have been reported. In Cx3cr1-deficient mice, where atrophic lesions develop upon aging and light-challenge, Sennlaub et al. demonstrated that the degeneration of the retina leads to CCL2 expression recruiting CCR2^+^ monocytes from the systemic circulation [38]. These CCR2^+^ monocytes infiltrate, accumulate, and participate in development of retinal atrophy; and the authors showed that pharmaceutical inhibition of the CCL2-CCR2 axis halts atrophy [38]. Studies on human patients with GA confirm the involvement of the immune system [24, 29, 39, 40]. Monocytes in patients with GA express CD200 more than healthy aged individuals, and patients who experience a fast progression of their atrophy have a higher CD200 expression level [40]. The glycoprotein CD200 is a ligand to the CD200 receptor (CD200R), and the CD200-CD200R interaction between peripheral monocytes and retinal microglia is thought to play a role in modulating neuroinflammatory activity [41–44]. Interestingly, in this study we find that the gene CD200R1, which encodes the CD200 receptor CD200R, is increasingly expressed in patients with GA, which further suggests the involvement of neuroinflammation in GA and possible contribution to this from the systemic circulation. In addition to this, another interesting finding was reported by Faber et al. who found a higher proportion of aged, differentiated memory CD8^+^ T-cells in a group of patients with AMD which consisted of early AMD, GA, and neovascular AMD [45]. In line with these findings were the enrichment analysis in this study, which suggested most strong signals related to type I interferon pathway and memory T cell differentiation. In an in vitro setting, Juel et al. demonstrated that when activated T cells are cocultured with RPE cells, RPE cells increase their expression of chemokines, which provides one explanation for the complex interplay involving both the innate and the adaptive immune system [46]. Taken together, these findings collectively suggest that both the innate and the adaptive immune system may play a role in GA; however, the picture remains unclear and further studies are warranted to investigate immunity in human patients stratified according to clinical subtype.

Unlike GA, both experimental and clinical studies have extensively documented the important contribution of the systemic immune system in neovascular AMD. Experimental laser-based induction of CNV in mice show strong recruitment of peripheral immune cells, and the inhibition of this process lead to a smaller CNV-size indicating that these immune cells play an important role in this process [47–49]. Observational studies in human patients with neovascular AMD confirm the involvement of both the innate and adaptive immune system [20–31]. Findings include higher proportion of pro-angiogenic CD11b^+^ and CCR2^+^ monocytes [25], dysregulation of CXCR3 expression in T-cells [24], and a higher proportion of aged cytotoxic CD8^+^CD56^+^ T-cells [26]. The latter is an interesting phenomenon thought to be an immunological ageing phenomenon that is accelerated in patients with neovascular AMD [26]. Results so far suggest that fibroblast growth factor receptor-1 (FGFR-1) may be a ligand for CD56 [50, 51]. FGFR-1 is expressed in human specimens of neovascular AMD and its role in retinal disease is also validated in an animal model of retinal injury [52, 53]. Our enrichment analysis suggest the involvement of immune response and lymphocyte activation, which is in line with otherwise reported observations in human patients with neovascular AMD. Interestingly, when Grunin et al. investigated the transcriptome in monocytes of human patients with neovascular AMD, the highest DAVID enrichment score was attributed to leukocyte/lymphocyte activation [34]. In this aspect, we confirm the findings of the Grunin et al. study. However, our enrichment analyses also suggest a strong involvement of gas homeostasis, including oxygen gas homeostasis. Considering that there is, to some extent, an overlap in the immunological findings in patients with neovascular AMD and GA, these findings collectively give rise to an interesting hypothesis: is the clinical manifestation of late AMD a question of *how* an aged immune system is phenotypically tailored to handle the abundant reactive oxygen species in the aged and stressed retina? To answer this question, further experimental studies and functional immunological studies are warranted.

Although anti-VEGF therapy of neovascular AMD has dramatically changed the prognosis for the patients, some still develop subretinal fibrosis, which is considered the untreatable end stage of the disease. Contributing mechanisms for subretinal fibrosis remain incompletely mapped but shares common mechanisms with fibrosis elsewhere in the human body, such as the lung and the skin [54]. Histopathological studies ofsurgical specimens have reported presence of fibroblasts, endothelium, and activated inflammatory cells in relation to the lesions [54]. Singh et al. reported that 25-hydroxyvitamin D, which is a circulating vitamin acting as a steroid hormone that suppresses inflammation, oxidative stress, angiogenesis, and fibrosis, is significantly lower in the plasma of patients with neovascular AMD that has subretinal fibrosis compared to those without [55]. Lechner et al. sampled plasma from patients with neovascular AMD with and without subretinal fibrosis, and found increased levels of C3a, C4a, and C5a, which are complement fragments measurable after complement activation through the classical pathway, mannose-binding lectine pathway, and the alternative pathway [21]. Our results in this study show that a large number of genes are differentially expressed between those with and without subretinal fibrosis, indicating that a complex range of immunological activities may contribute to this process. Enrichment analyses gives another perspective on this broad immune activation when suggesting activities related to mRNA splicing as well as ubiquitin- and caspase-activity. Interestingly, we also found signals related to endothelial migration and interleukin-1 signaling; these findings are in line with mechanisms and findings reported by other groups suggesting that elastin-mediated choroidal endothelial cell migration may play a role [56] as well as interleukin-1 signaling that contribute to the inflammatory response upon tissue stress that eventually leads to fibrosis [28, 57]. However, studies specific to subretinal fibrosis are lacking, and our findings suggest that these aspects should be studied further in a specific context of *subretinal fibrosis*.

Unlike neovascular AMD, PCV is far from being extensively studied, and much less in known regarding possible pathological mechanisms. Kumar et al. recently published a mouse-model of PCV and demonstrated that expression of the protease HTRA1 in RPE lead to degeneration of elastin in choroid mimicking features of PCV, which then progressed through infiltration of immune cells [58]. Details and characteristics of these immune cells remains unmapped; however, Sasaki et al. found increased IL-4 levels in the aqueous humor of eyes with PCV and Yu et al. found that cultured PBMCs from patients with PCV increasingly secreted IL-4 upon PHA-stimulation [59, 60]. We recently found that patients with PCV have a lower percentage of regulatory T cells that are increasingly Th2-like polarized [61]. Additionally, in patients with PCV, we recently found significantly higher plasma levels of IL-33 [61], which is a contributor to Th2-like polarization. Halim et al. mapped Th-like regulatory T cells and found that Th2-like regulatory T cells have increased migratory ability and higher viability and blasting capacity, which the authors linked to increased STAT5 phosphorylation that is known to promote angiogenesis [62, 63]. In this study, PBMCs of patients with PCV showed many differentially expressed genes related to T cell receptors and our enrichment analysis suggested involvement of immune effectors cells and Th2, as well as VEGF-activated receptor activity; which further suggests the involvement of recruited immune cells in PCV and that Th2 related immunity may be an important contributing factor.

Genetic susceptibility is a strong risk factor for developing AMD [4, 64, 65]. Heritability of late AMD was previously investigated in a twin study and estimated to 71% [64]. A range of single nucleotide polymorphisms (SNPs) has been identified that indicate involvement of the immune system with strongest associations in SNPs related to the complement pathways [4, 64, 65]. These findings are interesting in light with recent findings by Schemeidel et al. who demonstrated that SNPs associate with specific expression patterns in protein-coding as well as non-coding RNA regions in different immune cell types [66]. Hence, there may be an important link between the strong genetic susceptibility associated with AMD development and the transcriptomic profile of specific immune cell types which we find in this study that may contribute to disease development [66]. An interesting topic for a future investigation is to investigate the correlation between disease activity, genetic background, and the transcriptomic profile of specific immune cell populations. Comparison of the transcriptome of healthy retinae vs. retinae with AMD provide another important insight into the mechanisms of disease [67, 68]. In a transcriptomic study of retinae from eight pairs of postmortem retinae, Kim et al. found that the transcriptomic profile of retinae with AMD had alterations in pathways related to regulation of protein translational, mTOR signaling, phototransduction, and mitochondrial dysfunction [67]. The authors found differentially expressed anti-sense RNA, complement and apolipoprotein genes [67]. When looking at pathways, the anti-sense expression pattern in AMD was involved in pathways related to apoptosis, mitochondrial function, and oxidative stress response [67]. In a larger study of 453 postmortem retinae, Ratnapriya et al. investigated different stages of AMD and found that pathways related to immune regulation and cholesterol metabolism were upregulated in late AMD [68]. The authors also identified altered expression of genes related to extracellular matrix stability and protein degeneration [68]. Taken together, these findings underscore that AMD is a disease with a complex pathogenesis that involve local and systemic factors.

### Study limitations

When interpreting the results of this study, important limitations must be considered. This is a cross-sectional observational study; hence, we can only speculate on whether correlations identified in this study are indicative of a causal relationship. Experimental studies are needed to confirm any causality. In line with this limitation, it is important to understand that this study is exploratory. Our sample tissue was PBMCs, which consists of a range of immune cells with different functions. Another approach could be to focus specifically on individual cell types, which would give more detailed insight into mechanisms in specific cells. The gradient centrifugation-based separation of human blood to isolate PBMCs was performed at 4 °C, which is our standard setting for handling human blood. The manufacturer recommends room temperature, which yields a good compromise between time of separation (which is longer with lower temperatures) avoiding aggregation of erythrocytes which decreases the yield of lymphocytes (which is increased at higher temperatures). When we developed our protocols, we found satisfactory results upon centrifuging at 30 min (longer than the recommended 20 min) at 1000 *g* (faster than the recommended 800 *g*). However, it is important to note that this approach deviates from the recommended protocol by the manufacturer. Confirming and validating our findings warrant additional studies, e.g. confirmatory qPCR expression studies, testing of protein levels, and functional immunological studies. These studies could further establish specific roles in pathology and their implications. Finally, although we sampled an elderly healthy control group to better match the patient group (mean age 72 years), our groups of patients with GA (mean age 80 years) and patients with neovascular AMD (mean age 78 years) were significantly older. However, we accommodated to this issue with statistical adjustments.

## Conclusions

Increasing evidence find involvement of PBMC in late AMD. In this study, we present the global gene transcriptome of such PBMC across the different clinical subtypes of late AMD. We find that clinical subtypes of late AMD differ in PBMC gene expression profile. Our data support the findings of previous studies in patients with AMD and give rise to additional topics that warrants further investigation.

## Additional files

Additional file 1: Gene annotation list. (XLSX 1932 kb)

Additional file 2: Complete data of gene expression comparison across the three subtypes of late AMD. (XLSX 2968 kb)

Additional file 3: Heatmaps of differentially expressed genes in the three subtypes of late AMD. (DOCX 7945 kb)

Additional file 4: Complete data of gene expression comparison between neovascular AMD with and without subretinal fibrosis. (XLSX 1443 kb)

Additional file 5: Heatmaps of differentially expressed genes between neovascular AMD with and without subretinal fibrosis. (PNG 189 kb)

Additional file 6: List of significantly differentially expressed genes between neovascular AMD with and without subretinal fibrosis. (XLSX 47 kb)

## Data Availability

The datasets used and/or analysed during the current study are available from the corresponding author on reasonable request.

## Abbreviations

AMD: Age-related macular degeneration
ANCOVA: Analysis of covariance
BMI: Body mass index
BP: Biological process
CC: Cellular component
CD200R: CD200 receptor
CNV: Choroidal neovascularization
CS: Combined score
EDTA: Ethylenediaminetetraacetic acid
FC: Fold change
GA: Geographic atrophy
GO: Gene ontology
ICGA: Indocyanine green angiography
IQR: Interquartile range
MF: Molecular function
OCT: Optical coherence tomography
PBMC: Peripheral blood mononuclear cells
PCV: Polypoidal choroidal vasculopathy
RNA: Ribonucleic acid
RPE: Retinal pigment epithelium
SD: Standard deviation
VEGF: Vascular endothelial growth factor
