# Supplementary figures and images for: The transcriptome of peripheral blood mononuclear cells in patients with clinical subtypes of late age-related macular degeneration
Source: Immun Ageing. 2019 Aug 15;16:20. doi: 10.1186/s12979-019-0160-0 (PMC6696679; doi:10.1186/s12979-019-0160-0)

Patients with GA


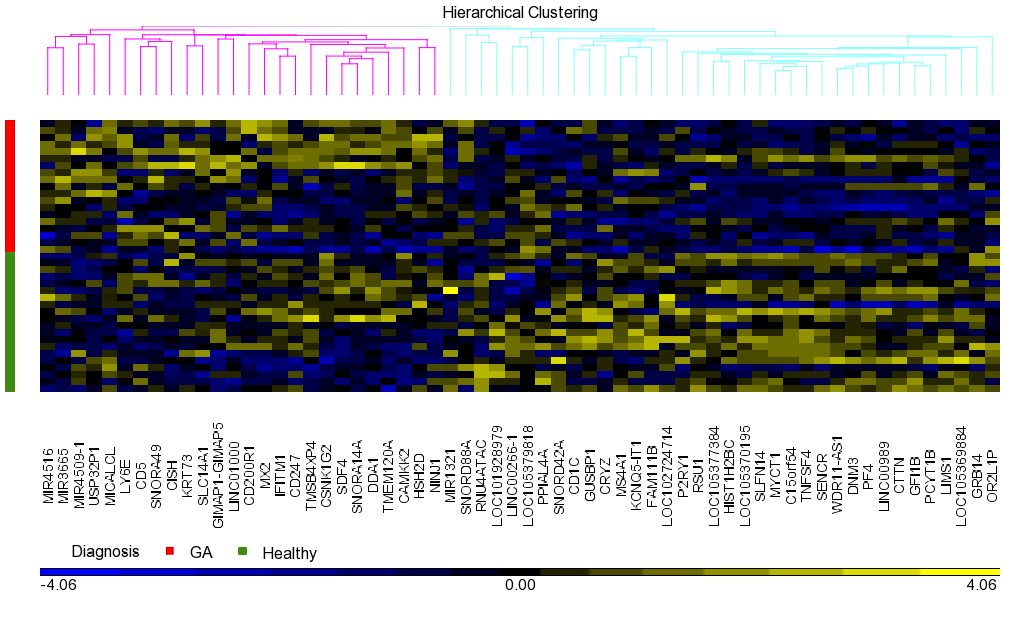


Patients with nAMD


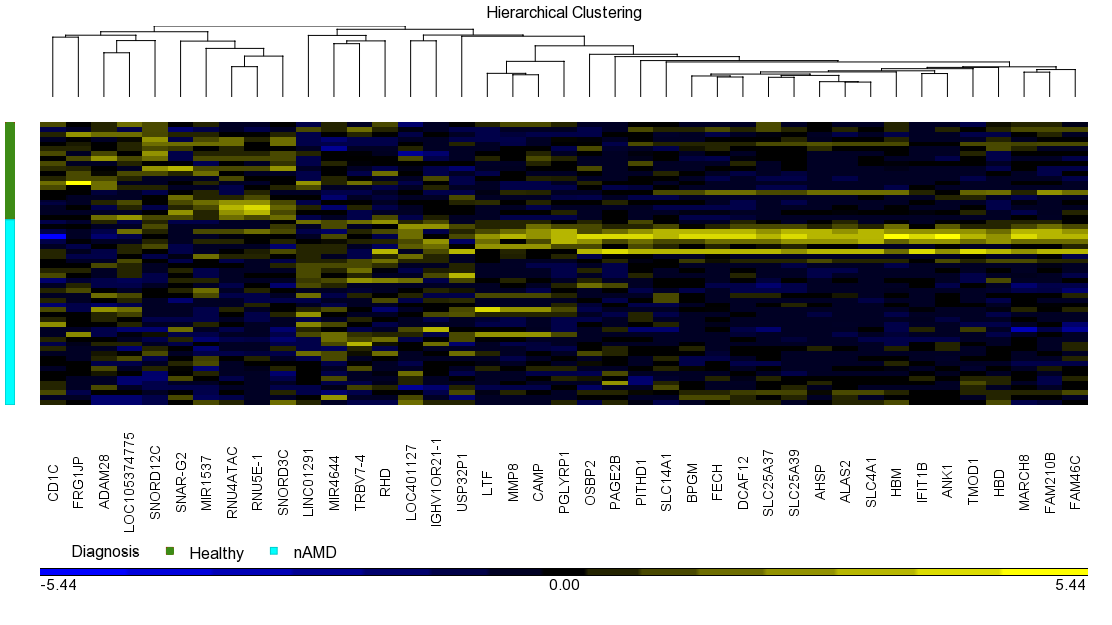


Patients with PCV


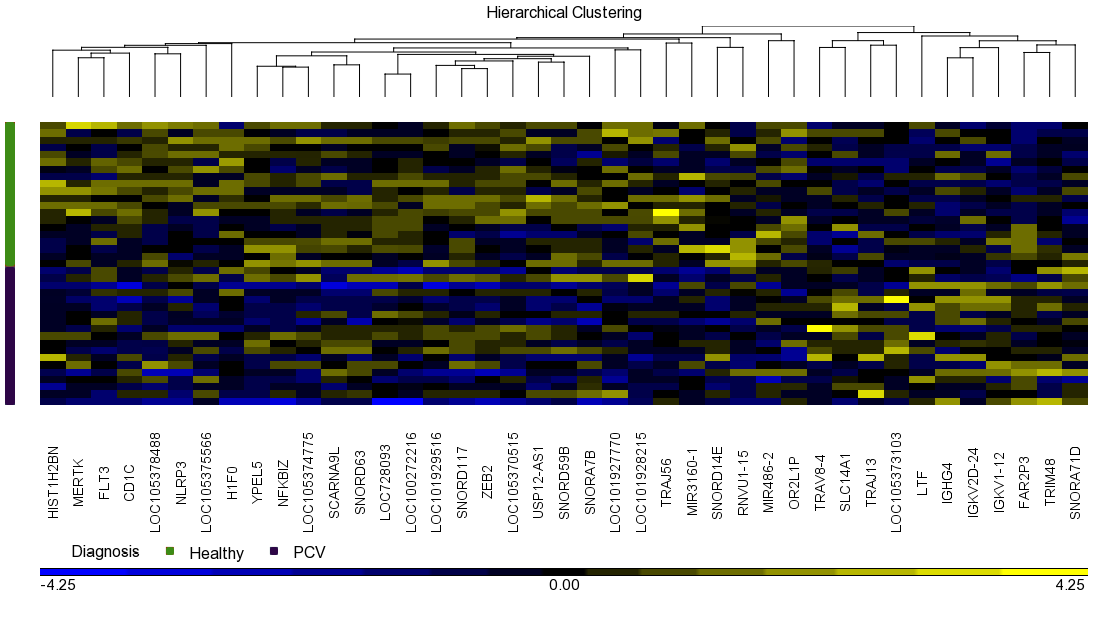

Supplement: Supplementary file 3 — Heatmaps of differentially expressed genes in the three subtypes of late AMD. (DOCX 7945 kb) [file 12979_2019_160_MOESM3_ESM.docx]

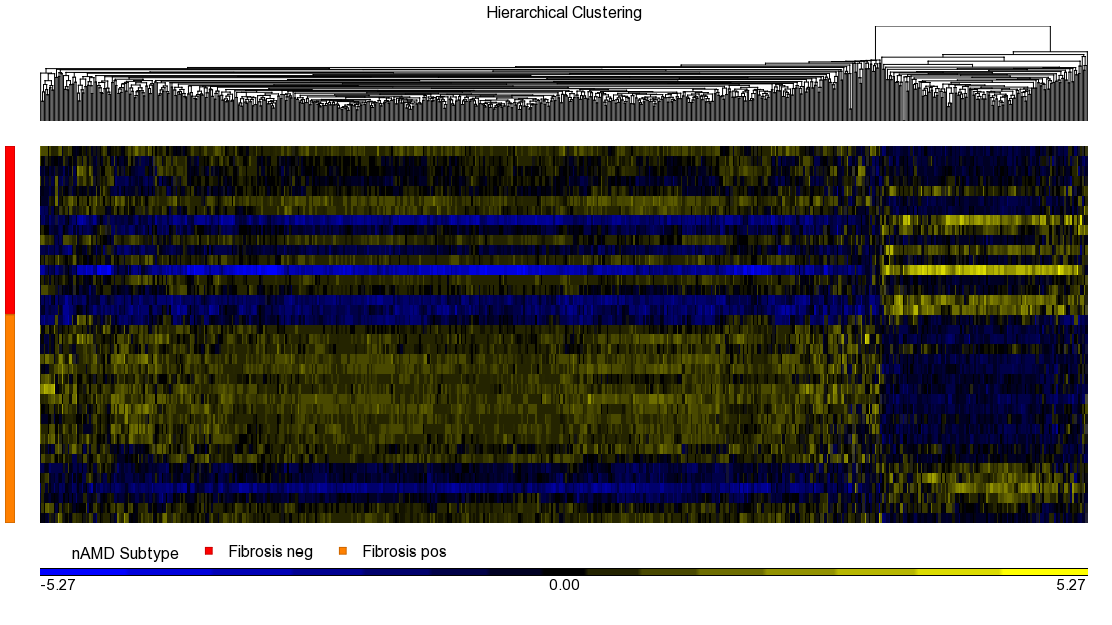

Supplement: Supplementary file 5 — Heatmaps of differentially expressed genes between neovascular AMD with and without subretinal fibrosis. (PNG 189 kb) [file 12979_2019_160_MOESM5_ESM.png]
